# Integrative Analysis of Molecular and Physiological Data to Quantify Biological Aging

**DOI:** 10.1093/geroni/igab046.117

**Published:** 2021-12-17

**Authors:** Daniel Belsky

**Affiliations:** Columbia University Mailman School of Public Health, New York, New York, United States

## Abstract

Development of reliable and valid measurements to quantify biological aging is a critical frontier geroscience. Originating in accumulations of molecular changes, biological aging undermines resilience within cellular networks and organ systems, driving disease, disability, and mortality. Measurements of biological aging have been proposed at several molecular and physiological levels of analysis. But agreement between measures implemented at different levels of analysis is low. The timing at which aging processes manifest at different levels of biological organization may vary, with the result that signs of aging manifest in one level of analysis are not yet observable in another. And different aging processes may be most apparent in different molecular levels of analysis. In midlife humans, aging-related changes are manifest at multiple molecular and physiological levels, making this population ideal for development of measurements that integrate information across levels of analysis to more precisely quantify the state and pace of biological aging.

